# An Adaptive Handover Prediction Scheme for Seamless Mobility Based Wireless Networks

**DOI:** 10.1155/2014/610652

**Published:** 2014-12-04

**Authors:** Ali Safa Sadiq, Norsheila Binti Fisal, Kayhan Zrar Ghafoor, Jaime Lloret

**Affiliations:** ^1^Faculty of Computer Systems and Software Engineering, Universiti Malaysia Pahang, Lebuhraya Tun Razak, Gambang, 26300 Kuantan, Pahang, Malaysia; ^2^UTM MIMOS CoE in Telecommunication Technology, Faculty of Electrical Engineering, Universiti Teknologi Malaysia (UTM), 81310 Johor Bahru, Johor Darul Takzim, Malaysia; ^3^Faculty of Engineering, Koya University, Danielle Mitterrand Boulevard, Koya, Kurdistan Region, Iraq; ^4^Instituto de Investigación para la Gestión Integrada de Zonas Costeras, Universidad Politécnica de Valencia, 46022 Valencia, Spain

## Abstract

We propose an adaptive handover prediction (AHP) scheme for seamless mobility based wireless networks. That is, the AHP scheme incorporates fuzzy logic with AP prediction process in order to lend cognitive capability to handover decision making. Selection metrics, including received signal strength, mobile node relative direction towards the access points in the vicinity, and access point load, are collected and considered inputs of the fuzzy decision making system in order to select the best preferable AP around WLANs. The obtained handover decision which is based on the calculated quality cost using fuzzy inference system is also based on adaptable coefficients instead of fixed coefficients. In other words, the mean and the standard deviation of the normalized network prediction metrics of fuzzy inference system, which are collected from available WLANs are obtained adaptively. Accordingly, they are applied as statistical information to adjust or adapt the coefficients of membership functions. In addition, we propose an adjustable weight vector concept for input metrics in order to cope with the continuous, unpredictable variation in their membership degrees. Furthermore, handover decisions are performed in each MN independently after knowing RSS, direction toward APs, and AP load. Finally, performance evaluation of the proposed scheme shows its superiority compared with representatives of the prediction approaches.

## 1. Introduction

Promising applications provided by emerging wireless networks are preferable as long as they can offer uninterrupted service during mobile node's (MN) roaming between access networks. Besides, the support of an efficient mobility management is considered one of very important issues for the future generation of wireless and mobile networks and services [1]. As expected, the production of wireless networks IEEE 802.11 which is known as wireless fidelity (WiFi) devices reached nearly 1.1 billion in 2011, which is also predicted to be doubled by 2015 [2]. Therefore, it is quite challenging to provide Internet connection with high quality-of-service (QoS) as a way to supply the running applications such as video, voice-over-IP (VoIP), navigation, and traffic monitoring. Normally, when MN roaming among access link candidates which offer different QoS level, MN must be able to chose the most appropriate network candidate to camp on. This can be achieved by obtaining the good network prediction technique with low handover delay which can support the desired QoS of ongoing applications [3–5].

Basically, network access link prediction is performing its processes during the time of handover decision making (wireless channel scanning period). This process started by performing the passive and active scanning and then selecting one network candidate as a way to perform the handover. In handover decision making system, the link layer handover process is required to be completed by the time known as a link layer delay. Thus, the handover signaling processes that are used for obtaining the new IPv6 address from visited network (network layer handover procedure) must wait until the link layer handover has performed its own process. Accordingly, the initiation time is increased afterwards, the overall latency will be increased as well. This can be considered as a main reason of degrading the QoS in WLANs [6, 7]. Therefore, the accurate handover prediction based on link quality and mobility aspects is challenging to achieve a seamless mobility with low handover delay [4, 8–12].

For this reason, there is a pressing need to develop an adaptive prediction technique in order to predict the most qualified AP. An adaptive fuzzy logic system has been proposed in order to address the issues of handover processes within wireless networks. Thus, the handover in link layer can be performed in predictive mode with low delay. The elaborated metrics, including received signal strength, mobile node relative direction towards access points in the vicinity, and access point load, are considered inputs of the fuzzy decision making system in order to select the best preferable AP around wireless local area network (WLAN). The obtained handover decision, which is based on the calculated quality cost using fuzzy inference system, is based on adaptive instead of fixed coefficients. In other words, the mean and standard deviation of the normalized fuzzy inference system's input metrics are applied as statistical information to adjust or adapt to the coefficients. In addition, this paper proposes an adjustable weight vector concept for input metrics in order to cope with the continuous, unpredictable variation in their membership degrees. Furthermore, handover decisions are performed in each MN independently after RSS, direction toward APs, and AP load are determined.

## 2. Related Work

One of the essential issues in wireless communications is the handover delay. Hence, many studies have tried to come out with appropriate solution to decrease the associated time delay during handover processes. For this reason, the prediction of next wireless network link with best QoS is highly required, which can be achieved by decreasing the handover delay issue. A number of studies that have been published earlier proposed several handover prediction techniques for WLANs. Some of these techniques are based on link quality aspects, whereas the mobility aspects are considered in the other techniques in order to obtain the handover decision. Therefore, this section tries to present the related works that were elaborated to improve the handover prediction for AP selection in WLANs.

In order to achieve the desirable QoS of ongoing applications, a handover decision making process based on predictive decision concept is proposed by [13]. A fuzzy logic based handoff decision algorithm was proposed in this study for maintaining the handover decision within wireless networks. The decision making parameters were data rate, RSS, and mobile speed which have been selected as inputs for the proposed fuzzy-based system as a way to select the best candidate AP. A handover scenario was introduced to be performed between WiFi and global service for mobile (GSM). The output of the proposed fuzzy algorithm in this method was setted to be a parameter called AP candidacy value (APCV). Afterwards, APCV was defined as a real number in order to rank the value of the candidacy level of the APs in scanning range.

However, the proposed fuzzy logic algorithm in [13] did not cover some other aspects that can improve the handover decision accuracy. For instance, when an AP is overloaded with many associated MNs, this can lead to a handover failure. Moreover, the authors did not consider the adaptation concept of membership functions of the input parameters in their method, which plays effective roles to maintain the handover decision under different circumstances of wireless network quality changing. In other words, the adaptable membership functions in the proposed fuzzy logic algorithm were not considered to maintain the adaptability in fuzzy handover system. Furthermore, in the proposed fuzzy logic inference system, the authors did not maintain a weight vector technique giving more impact to the parameter that has more variance behaviour compared to the other inputs.

The authors in [14], introduced dual-mode handsets and multimode terminals that are generating demand for solutions that enable convergence and seamless handover across heterogeneous access networks. Besides, the fuzzy logic approach has been proposed by [15], in order to handle the handovers between WLANs and universal mobile telecommunication systems (UMTS). The current RSS, Predicted RSS, and the bandwidth have been fuzzyificated and normalized to be used in handover decision making. This proposed fuzzy logic system reduced the number of handovers between WLANs and UTMS during MN roaming. Yet, in [15], the performance evaluation criteria such as handover delay, AP load, MN related direction towards each AP, and MN velocity are not addressed as key parameters in the proposed fuzzy logic system. In other words, by considering such key parameters, the probability of handover success will increase.

In contrast, the authors in [2] proposed a predictive fuzzy logic controller to reduce the channel scanning process. The proposed fuzzy system was designed based on Mamdani-type as a way to predict the next AP from a group of available APs obtained from scanning processes. Two input parameters utilized by the proposed fuzzy system are the average signal intensity (ASI) and the signal intensity variation (SIV). The ASI input is calculated at two-second intervals from the time beacon signal received by the MN, which is normally broadcasted by APs with an interval of 100 milliseconds.

However, the proposed predictive scheme basically relies on ASI metric that is normally used by MNs to estimate the need of performing the handover with available APs. Moreover, the SIV is elaborated to demonstrate the behaviour of the direction of MN towards available APs. Therefore, it can be observed that the obtained handover prediction using proposed scheme by [2] totally relies on the intensity of received signal from available APs. Thus, the probability of experiencing unnecessary and wrong handover prediction is high due to unpredictable movements and different channel propagation aspects. For instance, by the time the ASI and SIV are calculated and the handover decision has been triggered with an AP having the maximum values, the direction of MN is changed. This can yield connection breakdown with the new obtained AP due to the new movement direction that is unrelated to this AP; then ASI and SIV values started decreasing. To this end, the author in [2] could not efficiently address the issue of handover prediction within IEEE 802.11 WLANs.

Whereas, in [16], the authors proposed the Doppler frequency and a fuzzy logic system in the handover decision algorithm called the Adaptive Fuzzy Logic Based Handover Algorithm for Hybrid Networks, their approach supposes that if the MN speed is high, then triggering handover time will be decreased. Thus, avoid handover latency which is belonging to handover procedure. On the other hand, when MN speed is low, the trigger handover time will be increased to get more suitable networks. On the contrary, the proposed algorithm does not consider MN speed suitability to the next APs with different wireless technologies. Therefore, when the speed is high, the handover will fail. Moreover, the algorithm does not consider the load of each AP which leads to a handover failure as well.

In [17], the authors focused on the handover in AP dense 802.11 networks. Through this study, the AP scanning process has been highlighted in order to achieve an improved scan technique for 802.11 networks. Two key features, probe response arrival time and AP signal quality, were discovered in this study in a way to reduce the active probing time. Moreover, an improved version of D-Scan has been proposed by the aforementioned authors. The authors focused, in the first stage, on the probing wait time which is the MaxChannelTime. The authors decreased the active probing time by identifying the correlation between probe response arrival time and RSS quality. In this proposed approach, the handover is triggered based on the link quality of the current associated AP. It also performed a regular detection of the link quality of current AP. Whenever the current link quality of associated AP is poor enough which means that the handover is needed, that is, RSSI < HANDOFF-THRESHOLD, an actual handover process is enforced. Otherwise, if it is lower than a certain threshold (SCAN-THRESHOLD), the network interface card (NIC) is started to perform the background prescan. Eventually, all obtained APs are stored in a local AP database. Thus, the scan process was trying to find a certain number of APs with acceptable RSSI (>−75 dBm). When D-Scan process cannot find good Aps' RSSI quality on the current channel, it is switched to the next channel to scan until the whole frequency has been searched. Afterwards, the handover will be initiated with the AP that has more signal quality compare with others.

However, the authors in this study did not consider a smart prediction technique in the proposed D-Scan approach which can give high impact in handover process. Moreover, the D-Scan approach relies on the link quality of associated AP by monitoring the RSSI that cannot be reliable in APs-dense wireless networks due to fluctuations normally occurring during MN movement. Thus, the RSS value of collected APs independently changes; then, the handover decision obtained based on one metric (RSS quality) will be inaccurate.

## 3. Proposed AHP Scheme Overview

Fuzzy logic based mechanisms perform well in decision making systems, control, estimation, and prediction processes. For instance, in [18], Shih et al. proposed a production inventory model to precisely estimate seasonal demand and total demand. Other researchers in [19] utilized fuzzy logic in parallel interference cancellation (FLPIC) for frequency-selective fading channels in wireless CDMA communication systems. In addition, in [20], the authors used fuzzy logic in geographical routing when making packet forwarding decisions. In light of these applications, fuzzy logic has been applied in this study to select the most qualified AP in terms of RSS, MN direction, and AP load based on WLAN.

**Figure 1:**
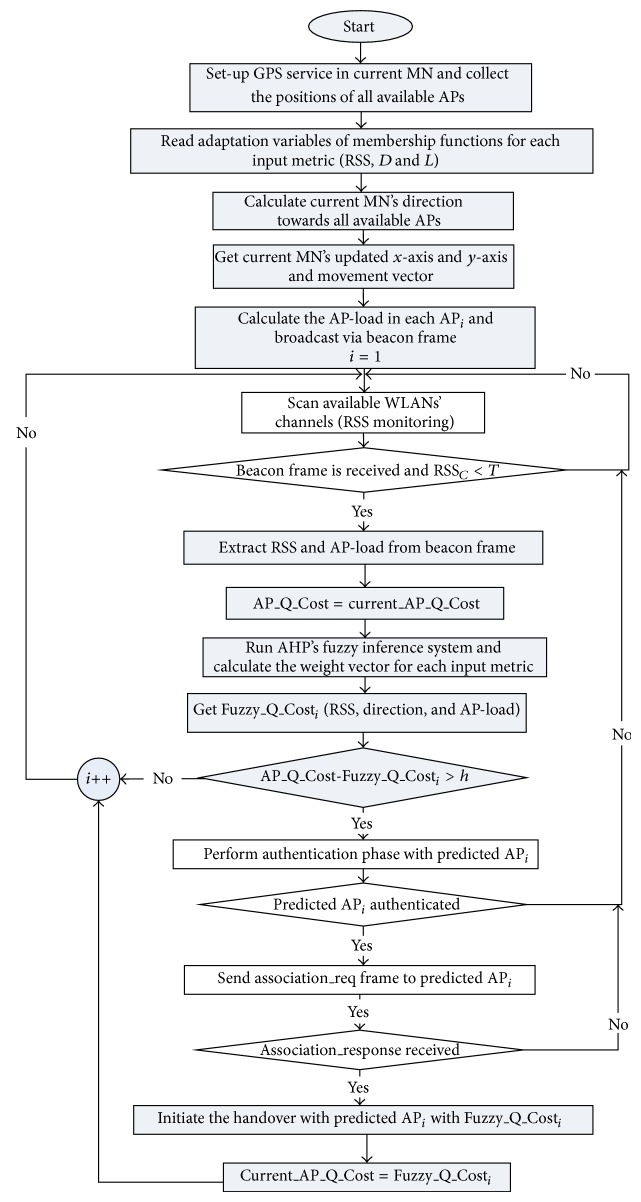
Flowchart steps of the proposed AHP scheme.

Figure 1 illustrates the systematic architecture of AHP design, implementation, and evaluation phases of the AHP scheme. It can be seen that the AHP's process begins with the collection of fuzzy inference's input parameters. In other words, in the first turn, the GPS set-up process will be performed in each MN in order to obtain the *x*-axis and *y*-axis of each AP in the simulated scenario along with updated MN's movement vectors. From the obtained data, the direction angle can be calculated in such a way as to observe the current MN's direction in relation to each AP in the roaming area. It should be noted that RSS monitoring is on whenever the WLAN's interface is “on” in order to ensure the highest quality of each available AP. This process is performed via wireless channel's passive and active scanning for the three nonoverlapped IEEE 802.11b channels (Channels 1, 6, and 11).

Furthermore, the current load of each AP is calculated and broadcasted via beacon frame. The RSS and AP load values will be extracted from the beacon frame of each AP within scanning range. After MN measures the received RSS of the current associated AP, the value RSS_*C*_ is compared with threshold value *T*. When RSS_*C*_ is less than *T*, the AHP algorithm begins the process of obtaining the handover decision for the next predicted AP candidate.

Therefore, the proposed AHP's fuzzy inference engine was utilized to obtain the quality cost of each collected AP *Fuzzy*-*Q*-*Cost*_*i*_. After the defuzzification process, the AHP checked whether *AP*-*Q*-*Cost* *of* *current* *AP*-*Fuzzy*-*Q*-*Cost*_*i*_ > *h*; if *yes*, the handover was initiated with selected AP_*i*_ and the *Current*-*AP*-*Q*-*Cost* was replaced with *Fuzzy*-*Q*-*Cost*_*i*_. *h* is the identified unnecessary handover restriction threshold which represents a quality cost threshold value. Should the obtained *Fuzzy*-*Q*-*Cost*_*i*_ exceed this value, the handover is not needed and afterwards restricted. Hence, based on AHP processes, the handover decision is taken with the AP with highest QoS, with consideration given at the same time to restrict unnecessary handovers.

### 3.1. Fuzzification of AP Selection Input Metrics and Output

As discussed previously, the selection criterion is one of the most challenging areas in the handover process, essentially affecting the handover delay in WLANs. Handover procedure should support always-best-connected (ABC) and always-best-satisfying (ABS) when selecting the target access station. Therefore, the lack of precise evaluation of the QoS metrics of the available WLAN candidates could be responsible for many of the drawbacks of ABC and ABS. For example, handover decisions which rely only on the quality of RSS for each AP selection process regardless of MN's direction in relation to each AP can actually increase the number of unnecessary or incorrect handovers. Similarly, when the selected AP currently serving many of the MNs is overloaded, handover failure phenomena may result. In other words, when the association request from the newly reached MN arrives at the overloaded AP, the probability that the association request will be discarded is quite high. Hence, in the following subsection, a fuzzy logic input metrics based AHP algorithm is discussed.

#### 3.1.1. Received Signal Strength RSS

Received signal strength RSS is one of the most common metrics used in handover decision making [10, 17, 21]. By monitoring RSS, the quality and distance of each AP in the range can be analyzed. When MN moves away from or towards an AP (ping-pong movement), the RSS for the AP will either increase or decrease. Therefore, during the passive scanning phase, the RSS in AHP scheme for each available AP will be captured and entered into the fuzzy inference system to be fuzzified. The range of RSS membership functions is considered to be in adaptive form. In other words, in order to achieve adaptive membership functions for RSS input metric, the RSS value to be distributed is to be between identified RSS_*Min*⁡_ and RSS_*Max*⁡_ normalized values. When the RSS for each AP in MN's scanning range has been collected during the passive scanning process, the RSS values are normalized and categorized using (1), (2), and (3).

**Figure 2:**
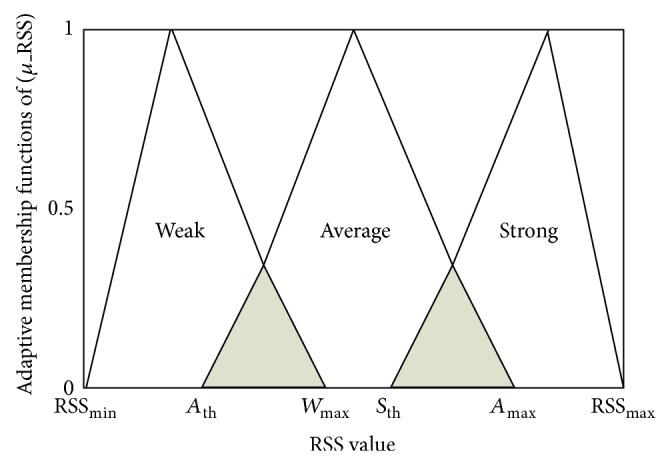
The adaptive membership functions of normalized RSS input metric.

Figure 2 shows the membership functions for RSS input metric. There are three RSS levels, identified as Weak, Average, and Strong, and the range of them was identified using the aforementioned assumed variables. By using variables, *A*_th_ threshold value of Average membership function, *W*_*Max*⁡_ maximum value of Weak membership function, *S*_th_ threshold value of Strong membership function, and *A*_*Max*⁡_ maximum value of Average membership function in addition to minimum and maximum values of RSS RSS_*Min*⁡_ and RSS_*Max*⁡_, the piecewise linear membership functions could be obtained as shown in (1), (2), and (3). By expanding these equations, the degrees between (0 to 1) of RSS's membership values are calculated, respectively, as shown in the vertical axis in Figure 2. Consider

(1) $$\begin{array}{c} \upsilon_{\text{RSS}}^{\text{Weak}}=\left\{\begin{array}{cc} 1, & \left(\text{RS}{\text{S}}_{\mathrm{Min}}\leq \text{RSS}\leq A_{\text{th}}\right),\\ \left|\frac{\text{RSS}-A_{\text{th}}}{W_{\mathrm{Max}}-A_{\text{th}}}\right|, & \left(A_{\text{th}}\leq \text{RSS}\leq W_{\mathrm{Max}}\right),\\ 0, & \left(\text{RSS}>W_{\mathrm{Max}}\right), \end{array}\right. \end{array}$$

(2) $$\begin{array}{c} \upsilon_{\text{RSS}}^{\text{Average}}=\left\{\begin{array}{cc} 1, & \left(W_{\mathrm{Max}}\leq \text{RSS}\leq S_{\text{th}}\right),\\ \left|\frac{A_{\text{th}}-\text{RSS}}{A_{\mathrm{Max}}-A_{\text{th}}}\right|, & \left(A_{\text{th}}\leq \text{RSS}\leq W_{\mathrm{Max}}\right)\\ & \text{or}\;\left(S_{\text{th}}\leq \text{RSS}\leq A_{\mathrm{Max}}\right),\\ 0, & \left(\text{RSS}>A_{\mathrm{Max}}\right)\;\\ & \text{or}\;\left(\text{RSS}<A_{\text{th}}\right), \end{array}\right. \end{array}$$

(3) $$\begin{array}{c} \upsilon_{\text{RSS}}^{\text{Strong}}\left\{\begin{array}{cc} 0, & \left(\text{RS}{\text{S}}_{\mathrm{Min}}\leq \text{RSS}\leq S_{\text{th}}\right),\\ \left|\frac{S_{\text{th}}-\text{RSS}}{\text{RS}{\text{S}}_{\mathrm{Max}}-S_{\text{th}}}\right|, & \left(S_{\text{th}}\leq \text{RSS}\leq A_{\mathrm{Max}}\right),\\ 1, & \left(A_{\mathrm{Max}}\leq \text{RSS}\leq \text{RS}{\text{S}}_{\mathrm{Max}}\right). \end{array}\right. \end{array}$$

Therefore, the proposed adaptive fuzzy system is used in the AHP scheme whereby the membership functions are identified adaptively. The RSS_*Min*⁡_, RSS_*Max*⁡_, *A*_th_, *W*_*Max*⁡_, *S*_th_, and *A*_*Max*⁡_ coefficients are designed in a way which can be set up by the user; thus, the membership's coefficients can be obtained adaptively. For instance, after identifying the value for each of the aforementioned coefficients (supposing −130, −10, −85, −70, −50, and −40 dBm, resp.), when the collected RSS value of an AP is −75 dBm, this value will be checked to identify which membership function it belongs to. Based on Figure 2 and after substituting the supposed values of each particular coefficient, the value −75 dBm is allocated in the triangle with rib of (*A*_th_, *W*_*Max*⁡_). In other words, when *A*_th_ = −85 and *W*_*Max*⁡_ = −70, (−85 ≤ −75 ≤ −70), hence, (1) is applied to range between 0 and 1. Thus, by substituting numerical values in |(RSS − *A*_th_)/(*W*_*Max*⁡_ − *A*_th_)|, it will be |((−75)−(−85))/((−70)−(−85))| = 0.66. This obtained value utilizing (1) implies that the collected RSS value of −75 dBm is allocated inside the weak membership function of RSS input metric and conflicts with medium membership function as well, so the degree of weak value is 0.66. Accordingly, the considered membership value is adaptively calculated for the input values neither totally inside nor outside any particular membership function.

#### 3.1.2. Relative Direction between MN and AP

The second fuzzy input metric is the related MN direction towards each AP. Basically, when an MN starts roaming across different Aps, it could determine more than one AP with a high quality RSS. On the other hand, this identified AP may not be situated in the same direction as the MN. In other words, the MN movement direction is not towards this particular AP. This scenario shows that the MN can obtain the wrong handover decision if the AP does not share the related direction with the MN. Therefore, the related MN direction towards each AP has been assigned as an input metric in the proposed AHP adaptive fuzzy inference system.

In simulation experiment scenario, the MN is equipped with a GPS system in order to obtain the updated *x*-axis and *y*-axis with every movement, in addition to the current MN's movement vector. The location of all APs in simulation scenario is defined as fixed positions in advance. When MN is in the first round, all AP positions will be collected through GPS navigator map, and the coordinates of these APs will be saved inside its own basic service set ID (BSSID). Thus, each time an MN roams through the network, the updated GPS map will be downloaded automatically from the server with *x*-axis and *y*-axis for all APs in the range and its own current position as well. With this process, the MN is aware of its own position in relation to the movement vector and the positions of APs in the range, which enables us to calculate the direction angle between the current MN position and each AP.

**Figure 3:**
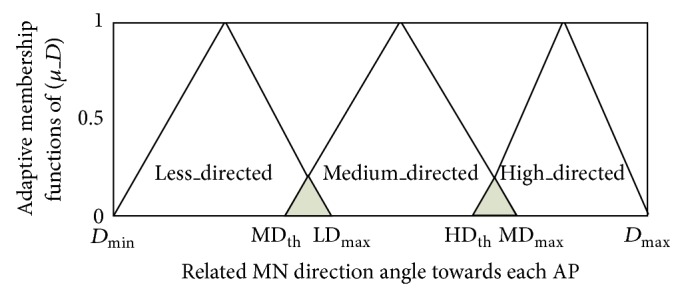
The adaptive membership functions of normalized related MN direction towards each AP input metric.

Formula (4) is used to calculate the direction angle's degree for each AP from the current MN position during its movement. Suppose that the current MN position is (MN_*X*1_, MN_*Y*1_), the position of AP is (AP_*X*2_, AP_*Y*2_), MN_*x*_ and MN_*y*_ represent the current position of the MN. Formula (4) describes how the direction angle has been calculated. The obtained crisp angle degree value will be entered to the fuzzy inference system as a second input parameter to be fuzzified with the other two inputs.  Figure 3 demonstrates the membership functions of related MN direction towards each AP input metric distributed as Less-Directed, Medium-Directed, and High-Directed.

The bearing angle (*θ*) between a MN and AP can be calculated as follows:

(4) $$\begin{array}{c} \cos\theta =\frac{{\text{MN}}_{x1}\cdot {\text{AP}}_{x2}+{\text{MN}}_{y1}\cdot {\text{AP}}_{y2}}{\sqrt{{\text{MN}}_{x1}^{2}+{\text{MN}}_{y1}^{2}}\cdot \sqrt{{\text{AP}}_{x2}^{2}+{\text{AP}}_{y2}^{2}}}. \end{array}$$

The range of membership function for directions selected to be between *D*_*Min*⁡_ which equals −1 reflects an MN directed less to one particular AP up to *D*_*Max*⁡_ which equals 1 and is highly directed towards AP. On the other hand, the variables LD_*Max*⁡_ Low-Directed membership function's maximum value, MD_th_ Medium-Directed membership function's threshold, MD_*Max*⁡_ Medium-Directed membership function's maximum value, and HD_th_ High-Directed membership function's threshold are identified in order to achieve adaptive direction membership functions. Using these identified variables, the coefficients of membership functions for direction input metric are obtained in an adaptive way. Utilizing (5), (6), and (7), the degrees of membership's values of direction metric are calculated based on identified coefficients.

For instance, when the identified coefficients in Figure 3 are set as *D*_*Min*⁡_ = −1, *D*_*Max*⁡_ = 1, MD_th_ = −0.4, LD_*Max*⁡_ = −0.2, HD_th_ = 0.6, and MD_*Max*⁡_ = 0.7, the obtained *D* value using (4) is 0.69 degree of *θ*. It is obvious that the *D* value of 0.69 is allocated in the highlighted triangle with the rib of (HD_th_, MD_*Max*⁡_) in horizontal axis in Figure 3 which is considered to be a conflict area between medium and high direction membership functions. Therefore, by applying (7)  (HD_th_ ≤ 0.69 ≤ MD_*Max*⁡_), the degree of high direction membership function is thus calculated to be in the range of 0 < Membership Degree < 1. Accordingly, by substituting the given coefficients in |(HD_th_ − *D*)/(*D*_*Max*⁡_ − HD_th_)|, the obtained degree is |(0.6 − 0.69)/(1 − 0.6)| = 0.225 High-Directed membership degree.

#### 3.1.3. AP Load

In order to achieve an accurate handover decision, a third input metric, the load in each AP, has been considered in the proposed adaptive fuzzy inference system of AHP. In some cases, the handover decision making mechanism could assign high quality cost to one AP which has a good RSS and is with a high direction angle's degree *θ* towards this AP. On the other hand, the selected AP might be overloaded. In other words, based only on the two aforementioned metrics with this particular AP and regardless of the number of MNs that are currently associated with it (by sending and receiving the traffic), the obtained handover decision is considered inaccurate. The implication is that when a new MN intends to establish a new handover process with an AP, the handover might fail due to the high load currently borne by that AP. For this reason, the AP load has been assigned as an additional input metric in the proposed adaptive fuzzy inference system of AHP in order to support an accurate handover decision.

To identify the membership function's range of AP load metric, a simulation experiment has been conducted. The outdoor campus of 500 ∗ 500 m is utilized to simulate the conducted wireless network scenario. In addition, the AP's transmit power and data rate are set at 60 milliwatt and 11 Mbps, respectively. Voice-over-IP traffic has been generated between MNs in the simulation area in order to test the load in the AP with real time applications. In order to precisely identify the maximum number of MNs that an AP can serve with reasonable throughput, the number of MNs is increased gradually in the simulated network area and the throughput has been collected in the AP side.

**Figure 4:**
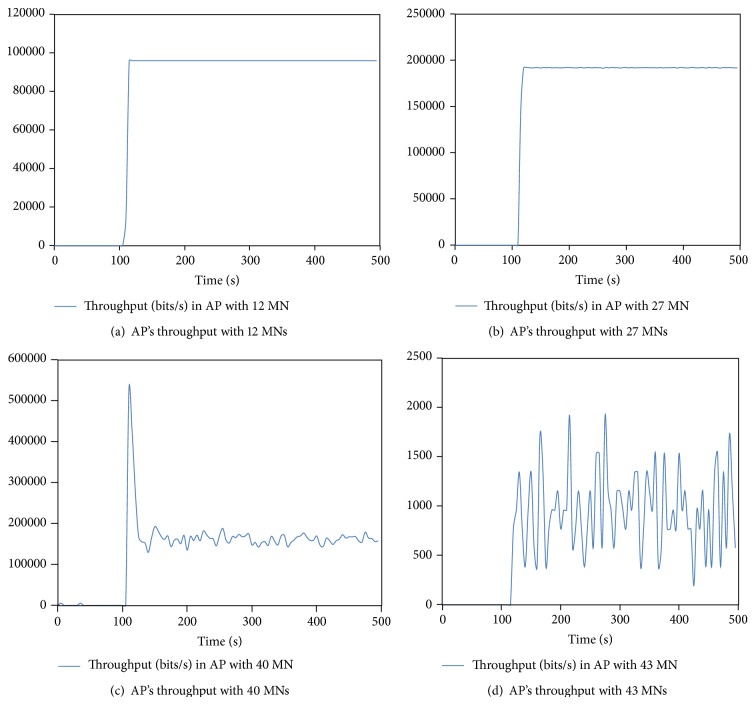
Analyses of the impact of increasing MNs number on AP's throughput.

The simulator experiment has been conducted four times and the AP's throughput is collected as shown in Figures 4(a), 4(b), 4(c), and 4(d). The AP throughput has been captured each time that MN's number increased.  Figure 4(a) shows that when the number of MNs was 12, the AP throughput after 105 seconds reached 96000 bits/sec with constant value until end of simulation. On the other hand, in Figure 4(b), the AP throughput was 192000 bits/sec when the number of MNs increased to 27. When the number of MNs reached 40 in Figure 4(c), the AP throughput after 110 seconds increased to 527424 bits/sec but rapidly decreased after 5 seconds to between 192768 and 145536 bits/sec. However, in Figure 4(d), when the number of MNs increased to 43, the AP throughput continued to decrease to the range of 192 to 1920 bits/sec. From this experiment, it can be concluded that the AP throughput with real-time traffic begins to decrease sharply when the number of associated MNs reaches 40. Consider

(5) $$\begin{array}{c} \upsilon_{D}^{\text{Less}}\left\{\begin{array}{cc} 1, & \left(-1\leq D\leq \text{M}{\text{D}}_{\text{th}}\right),\\ \left|\frac{D-\text{M}{\text{D}}_{\text{th}}}{\text{L}{\text{D}}_{\mathrm{Max}}-\text{M}{\text{D}}_{\text{th}}}\right|, & \left(\text{M}{\text{D}}_{\text{th}}\leq D\leq \text{L}{\text{D}}_{\mathrm{Max}}\right),\\ 0, & \left(D>\text{L}{\text{D}}_{\mathrm{Max}}\right), \end{array}\right. \end{array}$$

(6) υDMedium =1,LDMax⁡≤D≤HDth,MDth−DMDMax⁡−MDth,(MDth≤D≤LDMax⁡) or HDth≤D≤MDMax⁡,0,(D>MDMax⁡)or D<MDth,

(7) $$\begin{array}{c} \upsilon_{D}^{\text{High}}=\left\{\begin{array}{cc} 0, & \left(-1\leq D\leq \text{H}{\text{D}}_{\text{th}}\right),\\ \left|\frac{\text{H}{\text{D}}_{\text{th}}-D}{D_{\mathrm{Max}}-\text{H}{\text{D}}_{\text{th}}}\right|, & \left(\text{H}{\text{D}}_{\text{th}}\leq D\leq \text{M}{\text{D}}_{\mathrm{Max}}\right),\\ 1, & \left(\text{M}{\text{D}}_{\mathrm{Max}}\leq D\leq 1\right). \end{array}\right. \end{array}$$

Therefore, the AP load input metric range has been assigned to between *L*_*Min*⁡_ = 0 and *L*_*Max*⁡_ = 40 which represents the number of associated MNs. In other words, no MN currently associated with the AP indicates that the AP is currently with 0 load and is now “preferred.” On the other hand, when the number of MNs reaches 40, the AP currently with maximum load is “not-preferred.” The load range is normalized to be between 0 and 1 using the adaptation process for membership functions. Elaborating the received *S*_Count_ value from each AP, which is the Station Count collected from AP load elements in the beacon frame, the normalized AP load is obtained via adapted membership degree.

Basically, the AP_load_ value which consists the *S*_Count_ (number of associated MNs) is periodically broadcast via AP's beacons in the wireless network area. The AP load element in the AP beacon frame has been modified by adding a predetermined number ranging between 0 and 40.  Table 1 shows the modified AP load element in the beacon frame in each particular AP. Thus, when MN receives this amount of AP load, the adaptation process is applied in order to obtain the membership functions' degree of load input metric.

**Figure 5:**
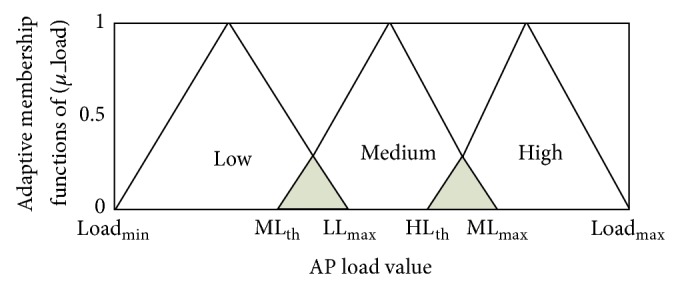
The adaptive membership functions of normalized AP load input metric.

Figure 5 shows the adaptive membership functions of normalized AP load input metric categorized as Low, Medium, and High. It can be seen that the variables ML_th_ medium load membership function threshold, LL_*Max*⁡_ low load membership function maximum value, HL_th_ high load membership function threshold, and ML_max⁡_ medium load membership function maximum value are identified in a way that allows for the calculation of the degree of each membership function. Equations (8), (9), and (10) are applied as a piecewise linear function to calculate the degree for each Low, Medium, and High membership function.

Similarly, a numerical example is illustrated in this paragraph in order to present the process of obtaining the membership functions' degree for AP load input metric. Suppose that ML_th_ = 0.35, LL_*Max*⁡_ = 0.4, HL_th_ = 0.73, ML_max⁡_ = 0.73, and the collected *L* value from an AP's beacon frame is 0.5. When the given value 0.5 is compared among the identified coefficients as presented in (8), (9), and (10), the appropriate membership function is subsequently selected. Thus, using (9), it can be observed that (LL_*Max*⁡_ < 0.5 < HL_th_) which implies that the given *L* value is completely under the Medium membership function. Therefore, based on the preceding equation, the value 1 is given as the input value of the medium membership function degree of 0.5*L*.

### 3.2. Design of Adaptive Fuzzy Logic for Handover Prediction System

The first step in designing a fuzzy inference system is to determine input and output variables and their fuzzy set of membership functions. An adaptive process is applied in order to obtain the degree of membership functions for each input metric. In addition, the adaptive weight vector is obtained by calculating the weight impact caused by the variance of each input metric and then determining the vector which helps to obtain the final fuzzy quality cost for each AP. This is followed by designing fuzzy rules for the system. Furthermore, a group of rules are used to represent the inference engine (knowledge base) to express the control action in linguistic form. The adaptive input metrics of the fuzzy inference system which are elaborated in AP selection and prediction process are presented in Section 3.1. Consider

(8) $$\begin{array}{c} \upsilon_{\text{Load}}^{\text{Low}}=\left\{\begin{array}{cc} 1, & \left(0\leq D\leq \text{M}{\text{L}}_{\text{th}}\right),\\ \frac{L-\text{M}{\text{L}}_{\text{th}}}{\text{L}{\text{L}}_{\mathrm{Max}}-\text{M}{\text{L}}_{\text{th}}}, & \left(\text{M}{\text{L}}_{\text{th}}\leq L\leq \text{L}{\text{L}}_{\mathrm{Max}}\right),\\ 0, & \left(L>\text{L}{\text{L}}_{\mathrm{Max}}\right), \end{array}\right. \end{array}$$

(9) υLoadMedium =1,LLMax⁡≤L≤HLth,MLth−LMLMax⁡−MLth,MLth≤L≤LLMax⁡, or (HLth≤L≤MLMax⁡)0,L>MLMax⁡ or L<MLth,

(10) $$\begin{array}{c} \upsilon_{L}^{\text{High}}=\left\{\begin{array}{cc} 0, & \left(0\leq L\leq \text{H}{\text{L}}_{\text{th}}\right),\\ \frac{\text{H}{\text{L}}_{\text{th}}-L}{L_{\mathrm{Max}}-\text{H}{\text{L}}_{\text{th}}}, & \left(\text{H}{\text{L}}_{\text{th}}\leq L\leq \text{M}{\text{L}}_{\mathrm{Max}}\right),\\ 1, & \left(\text{M}{\text{L}}_{\mathrm{Max}}\leq L\leq 40\right). \end{array}\right. \end{array}$$

#### 3.2.1. Adjustable Weight Vector for Input Metrics of Fuzzy Inference Engine in AHP

In this section, the process of obtaining the weight vector $\bar{W}$ which was calculated via (13) is presented. Taking into account various conditions, the values of each RSS, *D*, and *L* in addition to their membership degrees continuously vary in an unpredictable manner. In order to achieve the best handover decision under different conditions, the following features have been considered to obtain adaptable weight vector.

1. The weight vector for each input metric should not be fixed, meaning that it must be adjustable according to varying conditions.
2. The input metric whose value varies to a greater degree compared with other metrics, this metric is considered to be more important and it must have a higher weight.

For instance, assume that the RSS candidate value (MN_1_, MN_2_,…, MN_*n*_) has the maximum variance compared to the variance in the value of other metrics *D* and *L*. Therefore, it will be adjusted to the largest value in terms of weight vector. Equation (11) shows the calculation of the weight vector adjustment process for fuzzy input metrics. In this case, *A*_RSS_, *A*_*D*_, and *A*_*L*_ are the adjusted values of each input metric (RSS, *D*, and *L*), and *σ*_RSS_, *σ*_*D*_, and *σ*_*L*_ are the standard deviations of each input metric, respectively. Consider

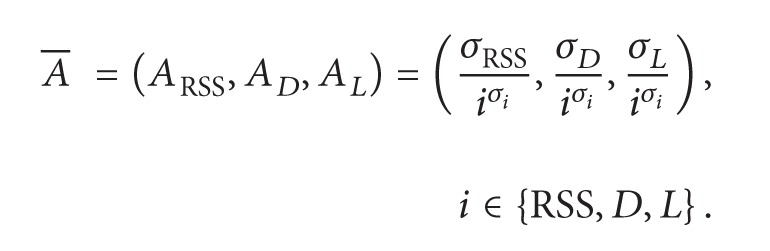
(11)


The standard deviation of the membership values have been normalized in (11), where the *σ*_*i*_ is the standard deviation of *υ*_*i*,1_, *υ*_*i*,2_,…, *υ*_*i*,*n*_ and *M*_*i*_ is their mean calculated utilizing the following equations (12). Consider

(12) $$\begin{array}{c} M_{i}=\frac{1}{n}n_{j=1}\upsilon_{i,j},\;i\in \left\{\text{RSS},D,L\right\},\\ \sigma_{i}=\bar{\frac{1}{n}n_{j=1}{\left(\upsilon_{i,j}-M_{i}\right)}^{2}},\;i\in \left\{\text{RSS},D,L\right\}. \end{array}$$

In addition, the RSS is considered a basic input metric for decision making in the handover process; thus, it should be highlighted that low variance of RSS metric should not be reflected in a decrease in its own weight vector. For instance, when the overall average of *υ*_RSS,*j*_  (*j* = 1,2,…, *n*) is low, the adaptation of membership degree of input metric RSS must be tackled more seriously. For this reason, the weight vector of RSS input metric, *W*_RSS_, should be given a higher weight value among the other two input metrics (*D* and *L*). Thus, the link-breakdown probability is reduced by ensuring that the handover decision based on RSS parameters during the time of link quality is weak, ranking value is in overall average. On the other hand, when the mean value of RSS *υ*_RSS,*j*_ is high in overall average, the effects of its weight vector will be moderated among the other two input metrics (*D* and *L*).

Moreover, a weight vector, $\bar{W}$, is identified which presents the weight of input metrics, RSS, *D*, and *L* as follows:

(13) $$\begin{array}{c} \bar{W}=\left[W_{\text{RSS}},W_{D},W_{L}\right]. \end{array}$$

The detailed weight vector calculation is presented using the following equation (14), whereas the average variance is tackled seriously with RSS input metric rather than other two input metrics (*D* and *L*), as presented in the example in the previous paragraph. The important point to note here is that (14) is adjustable based on the input metric that is more variable during the MN's roaming process. For instance, when the MN is moving with many changes in direction angle *θ*, the mean of direction input metric *M*_*D*_ will be considered in (14). Hence, the accuracy throughout this feature improves the handover decision making process. Consider

(14) W¯=(wRSS,wD,wL)=ARSSMRSS,MRSS×AD,MRSS×AL.

Suppose that, for the *j*th  (*j* = 1,2,…, *n*) mobile station, namely, MN_*j*_, the membership degree vector $\bar{U_{j}}$ has been defined from combination of three input metrics (RSS, *D*, and *L*). Consider

(15) $$\begin{array}{c} \bar{U_{j}}=\left\{\begin{array}{cc} \upsilon_{\text{RSS},j} & \\ \upsilon_{D,j} & \\ \upsilon_{L,j}. & \end{array}\right. \end{array}$$

Based on (15) and (13), the final fuzzy cost Fuzzy_Cost_*i*__ of mobile station *j*th can be obtained utilizing (16). Consider

(16) $$\begin{array}{c} \text{Fuzz}{\text{y}}_{\text{cost}}=\bar{U_{j}}\cdot \bar{W}. \end{array}$$

**Figure 6:**
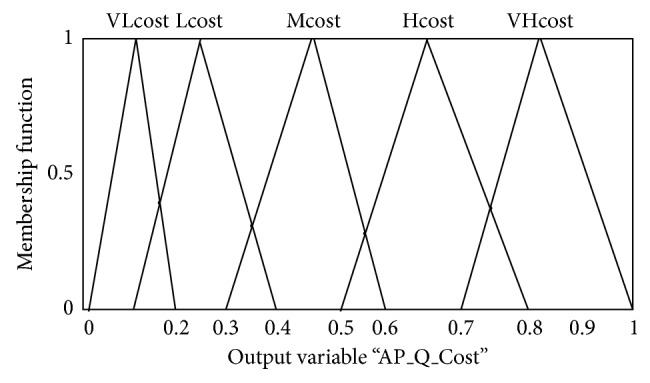
The membership function for AP quality cost output metric.

The output AP quality cost from fuzzy inference system Fuzzy_Cost_*i*__ is configured to range between (0 to 1 Rank) from lower value to the higher value of quality cost for each AP. For instance, Figure 6 shows that the division of quality cost output of AP has five levels of rank: VLcost, Lcost, Mcost, Hcost, and VHcost. The final fuzzy inference decision is based on the adaptive membership degree vector of each input metric and the weight vector as presented in (16). Moreover, triangular functions are used as membership functions as they have been widely used in real-time applications due to their simple formulas and computational efficiency. It is important to highlight that a good membership function design has a significant impact on the performance of the fuzzy decision making process.

#### 3.2.2. Adaptive Fuzzy Inference Engine

In the proposed AHP scheme, the adaptive membership function is proposed and utilized in the design of a fuzzy inference system. Moreover, it is important to mention that the precise design of membership function has a major impact on the overall performance of the fuzzy prediction process. Furthermore, the proposed weight vector concept and the best AP selection process contribute positively to increase the quality of the obtained final handover decision.  Table 2 demonstrates the utilized fuzzy rules in the proposed fuzzy inference system.

#### 3.2.3. Defuzzifcation

Defuzzification refers to the way that a crisp value is extracted from a fuzzy set value. In the proposed fuzzy decision making system in AHP, the centroid of area strategy for defuzzification has been considered. This defuzzifier method is based on Formula (17) as follows. Consider

(17) $$\begin{array}{c} \text{Fuzz}{\text{y}}_{\text{cost}}=\frac{\sum_{\text{All}\;\text{Rules}}^{}\bar{U_{j}}\times \bar{W}}{\sum_{\text{All}\;\text{Rules}}^{}\bar{U_{j}}}, \end{array}$$

where Fuzzy_cost_ is used to specify the degree of decision making, $\bar{W}$ is the weight vector variable of input metrics (RSS, *D*, and *L*), and $\bar{U_{j}}$ is their adaptive degree of membership functions. Based on this defuzzification method, the output of the AP-Q-Cost is changed to a crisp value.

### 3.3. The Best AP Selection Process in AHP

The handover decision is performed in local host mode as each MN measures the received RSS and its direction degree towards each available AP. In addition to the received AP load value, which is broadcast via each AP, the handover decision utilizing the implemented AHP (whenever RSS from current serving AP, RSS_*c*_, degrades below a threshold *T*) is then carried out. Consider

(18) $$\begin{array}{c} \text{RS}{\text{S}}_{c}<T. \end{array}$$

Afterwards, the decision factor, AHP_*F*_, based on the calculated Fuzzy_Q_Cost_*i*___ for all the candidates is obtained and the AP_*i*_ candidate is chosen for handover initiation if the following condition is satisfied:

(19) $$\begin{array}{c} \bar{{\text{AHP}}_{F}}=\text{A}{\text{P}}_{\text{Cost}}-\text{Fuzz}{\text{y}}_{{\text{Cost}}_{i}}>h, \end{array}$$

where *h* is the threshold value which helps to avoid unnecessary handovers, Fuzzy_Cost_*i*__ is the final decision metric of maximum quality cost of AP_*i*_ candidate, AP_Cost_ is the quality cost of the currently serving AP, and $\bar{\text{AH}{\text{P}}_{F}}$ is the difference between decision factor of the serving AP and the AP_*i*_ target.

## 4. Performance Evaluation

To evaluate the performance of the proposed AHP scheme, a simulation scenario is created employing OMNET++ simulator and the AHP scheme was implemented along with the state of the art, which are the existing AP prediction methods in wireless networks. The evaluation is conducted based on several metrics which are the impact of MN's number on average handover delay, impact of MN's number on average handover delay, AP load, total number of handovers, number of failed handovers, handover failure probability, average MAC-layer delay, the impact of MN's number on packet loss ratio, and adaptive Fuzzy-Quality-Cost of available APs in simulation scenario.  Table 3 demonstrates the simulation parameters that were utilized in simulation AHP scheme by OMNeT++.

In order to achieve simplicity in presenting the simulation results, the two compared methods are represented by short-form style. The method proposed in [13] is denoted as access point candidacy value (APCV), whereas the other method, in [22], Scan in AP-dense 802.11 networks is called D-Scan. On the other hand, adaptive handover prediction has been previously identified as an AHP scheme. A detailed discussion of all the aforementioned evaluation metrics is presented in the subsections below.

### 4.1. Impact of MN's Number on Average Handover Delay

Figure 10(a) illustrates the impact of MN's number on obtained average handover delay based on 5 simulation runs. As a function of MN's number increasing up to a maximum of 50 MNs, graphs of average handover delay in seconds are collected and presented for each AHP scheme and APCV and D-Scan methods in Figure 10(a). As can be seen, as the number of MNs is increased to 50, the AHP scheme performed the best in decreasing the overall average handover delay. More precisely, the handover delay with AHP scheme maintained an average of 0.06 to 0.09 sec, when the number of MNs increased from 1 to 18. In contrast, when the number of MNs increased to 19, the average handover delay increased to 0.1 sec. The handover delay kept increasing slightly, on average, as the number of MNs reached 22; the average delay was fixed to 0.23 sec up to 50 MNs.

On the other hand, the achieved average handover delay utilizing APCV method was very similar to the one obtained by AHP scheme with 10 MNs running in a simulated scenario. This delay started to increase sharply after 18 MNs. It can be observed from the resulting graph of APCV method that the average handover delay reached 1.098 sec when the number of MN's reached 43. However, the delay keep increasing similar to the increase which occurred in MN's number until reaching 2.84 sec with 50 MNs. In contrast, although D-Scan method is designed to decrease the handover delay by incorporating smart scanning processing in the link layer, a worse performance is observed with respect to both the AHP scheme and APCV method when the number of MNs is increasing. As observed from the results presented in Figure 10(a), note that the average handover delay began to increase sharply after 29 MNs (more than 1 sec delay), compared to both AHP and APCV results. The average obtained handover delay by D-Scan method continued to increase as the number of MNs increased until it is reached 2.99 sec after 43 MNs.

In fact, the serious improvement in decreasing average handover delay which was achieved using the proposed AHP scheme is due to the fact that the handover decision in the AHP scheme is obtained in cooperation with adaptive AP load input metric. Therefore, the handover process did not encounter any overloaded APs, keeping the average handover delay low regardless of the increase in the number of MNs. However, this feature was not considered in either the APCV or D-Scan method which resulted in the failure to reduce the handover delay in the low average range in response to increases in the number of MNs. Finally, it is worth mentioning that the AHP scheme could efficiently decrease the average handover delay as the number of MNs continued to increase. This was achieved by developing adaptive coefficients of the mean and standard deviation of the normalized fuzzy inference system's input metrics.

### 4.2. AP Load

As mentioned earlier, AP load is an important metric that must be considered during the handover decision making process. Handover decisions obtained with one particular AP with high load cause high handover delay and might cause handover failure. Hence, the AP load considered in the proposed AHP is an important metric that contributes positively to the AP rankings. This leads to making handover decisions with the most qualified AP candidate by taking into account its current load. Moreover, the AHP scheme using AP load metric made an essential contribution in support of wireless networks by creating load balancing among APs, ensuring or improving accuracy in handover decision making. Therefore, as can be seen from Figure 10(b), the AHP scheme reduced the load balance that was tackled by each AP and distributed it fairly among all 9 APs in the simulation scenario.

In order to provide a perspective example of calculated AP load, the average load obtained from AP1 is highlighted in this paragraph. Alternatively, Figure 10(c) presents the average of obtained AP load of AP1 utilizing AHP scheme and APCV and D-Scan methods measured in bits/sec during simulation time. As a function of load (bits/sec) tackled by AP1 during simulation time, the output graphs of AHP scheme and APCV and D-Scan methods over the first 100 seconds, all acting on the same load, are shown. The reason is that, during this period of time, AP1 is still serving only the first round of MNs. When the new MNs begin to associate with AP1 as a result of handovers beginning after 100 seconds, the obtained load by both APCV and D-Scan is increased sharply. At the same time, the load obtained by employing AHP scheme continued to decrease throughout the simulation time compared with APCV and D-Scan which obtained higher loads, respectively.

In percentage form, the AP1 load as presented in Figure 10(b) indicates that the achieved load is the lowest using the AHP scheme followed by D-Scan and APCV methods, respectively. Similarly, in Figure 10(c), the AHP scheme is superior in terms of decreasing the load (bits/sec) performed by AP1 during simulation time compared with the state of the art or the existing AP's prediction methods. This has a tremendous effect on the load balancing among the available APs in the simulated area. Thereby, the handover process avoids overloading APs as long as there are alternative APs with better quality cost obtained using the proposed adaptive fuzzy inference system.

### 4.3. Total Number of Handovers

**Figure 7:**
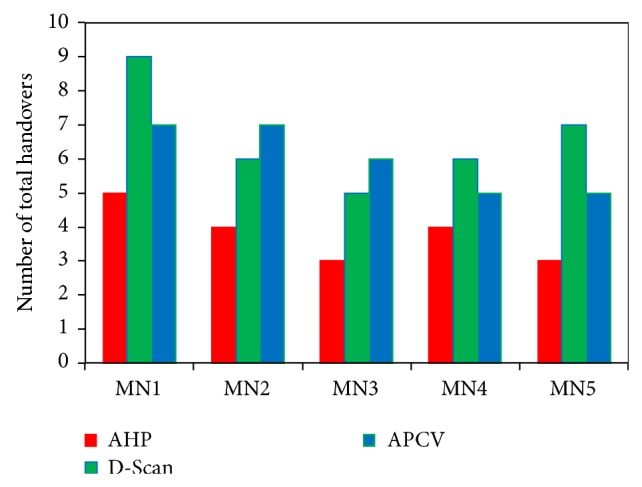
Number of total handovers.

In order to evaluate the proposed AHP scheme in terms of the ability to maintain the total number of handovers at an acceptable level, five MNs have been selected to observe the average number of handovers that are performed with each simulation run. Throughout this evaluation metric, the level of improvements in prediction accuracy can be studied and analyzed as a way to validate the proposed AHP scheme's performance. The total number of handovers processed during the simulation time by the five selected MNs has been captured and then calculated.  Figure 7 illustrates the total number of handover decisions triggered by each of the five MNs (successful and failure handovers) employing AHP, APCV, and D-Scan. It was observed that, by using the proposed APH scheme, the average number of handovers that are obtained by MN1 is 5, whereas MN2 and MN4 are achieved 4. On the other hand, the obtained average handovers within MN3 and MN5 was 3 handovers. Utilizing APCV and D-Scan, the average number of total handovers were 9, 6, 5, 6, and 7 and 7, 7, 6, 5, and 5 as a sequence of five selected MNs, respectively.

It is obvious that proposed AHP scheme performs better than both APCV and D-Scan methods in terms of reducing the total number of handovers. In other words, by using the AHP scheme, unnecessary and incorrect handover decisions have been significantly reduced or avoided. This is due to the fact that, in proposed AHP scheme, the MN calculates the quality cost of each neighbour AP using the proposed adaptive fuzzy inference system before performing the handover process. Thus, the obtained handover decision is based on the correlation between three fuzzified input metrics (RSS, related direction, and AP load) and is more accurate.

### 4.4. Number of Failed Handovers

**Figure 8:**
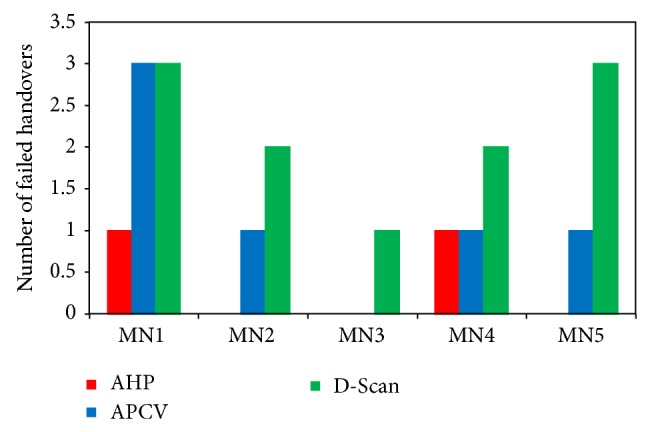
Number of failed handovers.

From another perspective, to evaluate the proposed AHP scheme in terms of reducing the number of unsuccessful handovers, the average number of failed handovers in each of 5 selected MNs has been calculated. Through conducting this performance test, the ability in obtaining correct handover predictions in WLANs can be examined, which subsequently contributes in reducing the handover delay. By looking at Figure 8, it can be observed that MN2, MN3, and MN5 using the proposed AHP scheme did not face any handover failure during simulation time. However, MN1 and MN4 obtained one handover failure. In contrast, the number of failed handovers in each of 5 MNs using both APCV and D-Scan was 3, 1, 0, 1, and 1 and 3, 2, 1, 2, and 3, respectively. In different form, when counting the average number of failed handovers out of the five MNs as presented in Figure 8 for the three applied schemes, the obtained average number using each implemented scheme was 0.4 AHP, 1.2 APCV, and 2.2 utilizing D-Scan. This indicates that the proposed AHP scheme achieved the lowest average of failed handovers while APCV and D-Scan methods followed in rank order. This is not surprising since the proposed AHP scheme relies on a predictive fuzzy inference system based on three input metrics (RSS, related direction, and AP load). Hence, the handover process was performed each time with the most qualified AP candidate in the scanning area.

On the other hand, in APCV method, the MN obtained the handover decisions with APs based on the candidacy value obtained via fuzzy logic regardless of AP's current load factor and its related direction aspect. In contrast, D-Scan method relies only in a predictive way on performing a fast and active scan for existing APs which contributes to reducing the Maximum Channel scanning time. The simulation experiment conducted in this regard shows that the D-Scan method achieves low total handover latency in comparison with APCV, while, at the same time, the number of failed handovers increased. This is due to the fact that the D-Scan method focused on performing scanning process in less time than obtaining the handover decision with an AP collected from APs list by comparing their RSS with the current AP. An additional weakness is that this type of decision making system can fall into inaccurate handover decisions easily. Alternatively, it can be concluded from Figure 8 that the proposed AHP scheme could achieve a low number of failed handovers in comparison with both APCV and D-Scan methods due to accurate handover decisions based on an adaptable fuzzy inference system.

### 4.5. Handover Failure Probability

**Figure 9:**
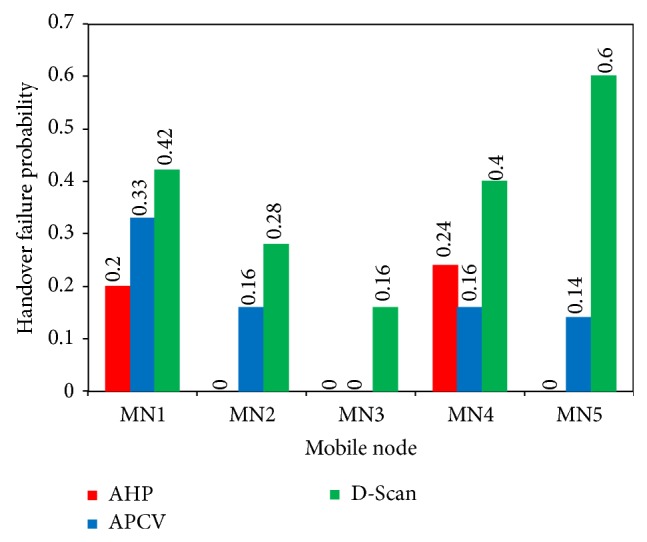
Handover failure probability.

The probability of handover failure in unit of zero (Low) to 1 (High) for the five selected MNs in the experiments is considered under this section. The simulation outcome of varying number of failed handovers using proposed AHP scheme in comparison to D-Scan and APCV methods was calculated to demonstrate the probability of failure. It is under such circumstances that the probability of handover failure can be calculated based on the mean of obtained failed handovers that were previously recorded. Handover failure probabilities have been calculated for each of the five selected MNs and are illustrated in Figure 9. In Figure 9, the probability of handover failure is shown in comparison form, and the probability of failure achieved by the AHP scheme, APCV, and D-scan MN1 is 0.2, 0.33, and 0.42, respectively. This implies that MN1 explained in the proposed AHP scheme could obtain the lowest failure probability compared with the other two methods. In MN2, the probability of failure was 0, 0.16, and 0.28, respectively, which shows that the AHP scheme achieved zero handover failure probability with MN2. The calculated failure probability for MN3 was zero in both AHP scheme and APCV while it was 0.16 in D-Scan method. On the other hand, APCV method with MN4 obtained 0.16 as the lowest failure probability in comparison with proposed AHP scheme at 0.24 and the D-Scan method at 0.4.

To sum up, the calculated failure probability in MN5 was zero by using AHP scheme, while it were 0.14 with APCV, and 0.6 with Scan method. It can be summarized from Figure 9 that the handover failure probability using the AHP scheme in MN1, MN2, and MN5 was the lowest compared with the other two methods. In contrast, the probability in MN3 was the same as AHP and APCV, which was zero probability. Last but not least, in MN4, the APCV achieved less failure probability compared with AHP and D-Scan. Therefore, in terms of the overall probability of handover failure, the AHP scheme is superior in comparison to the state of the art in reducing the probability of failure due to the aforementioned reasons.

### 4.6. Average MAC-Layer Delay

**Figure 10:**
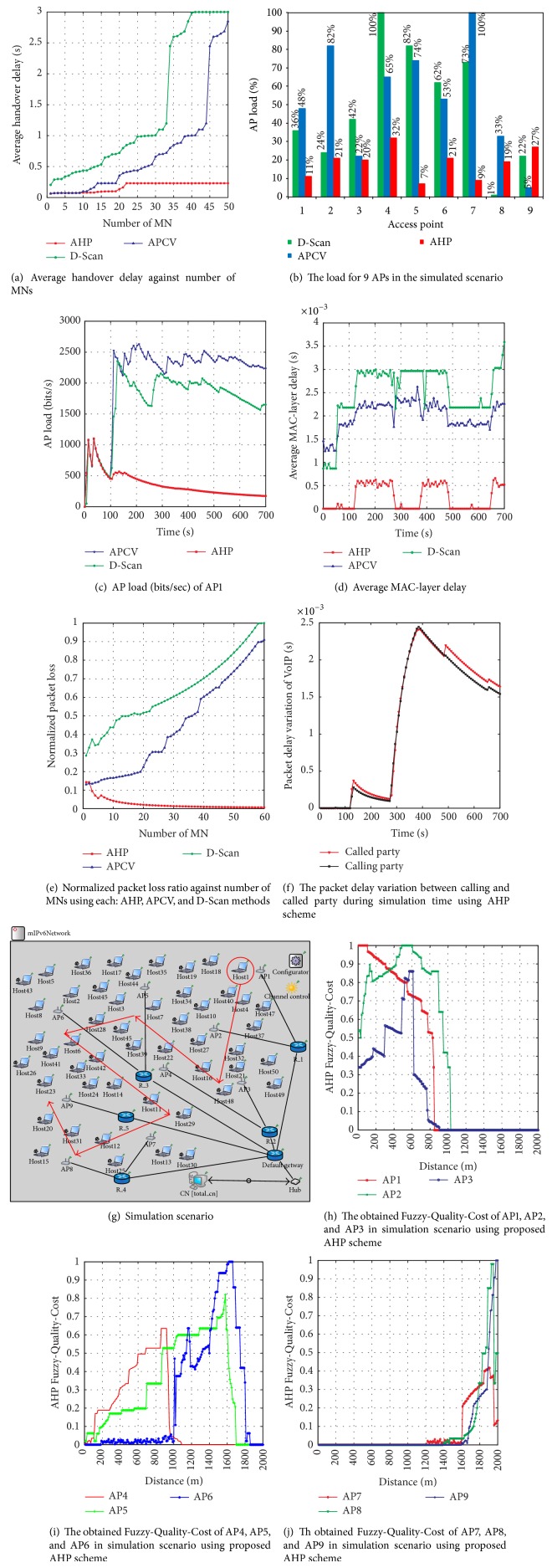
Performance evaluation.

Figure 10(d) shows the average MAC-layer delay (measured in seconds), in comparison form between the proposed AHP scheme, D-Scan, and APCV during simulation time. Generally, it can be observed that the proposed AHP scheme obtained the lowest average MAC-layer delay out of 5 simulation runs compared with APCV and D-Scan methods. The graph presented in Figure 10(d) illustrates the varying rate in MAC-layer delay with the impact of handovers which are performed during simulation time. Therefore, by looking at the fluctuations in the depicted graphs, the behaviour of the MAC-layer delay during the handover time is split between MN and APs in the simulated area. On the other hand, a straight line graph indicates that the MAC-layer is currently not in the handover process (MN is currently active with one AP).

In fact, the proposed AHP scheme performed handover decisions accurately, avoiding unnecessary handovers. As presented in Figure 7, the total number of handovers is less than that of the other two methods, and the MAC-layer delay is critically decreased. Hence, the MAC-layer delay, which is susceptible to high delay due to many handover processes, has been saved from having to do so many fluctuations as the AHP scheme reduces the number of handovers. In contrast, APCV method obtained higher delay compared with AHP scheme, since APCV does not consider any adaptation process or weight vectors in order to improve handover decision making in fuzzy inference systems. Subsequently, the delay increased during simulation time compared with the AHP scheme. On the other hand, the MAC-layer delay increased sharply after 50 seconds using the D-Scan method, being in the average higher than both APCV and AHP scheme. It should be noted that the D-Scan method focuses on smartness during the scanning process in MAC-layer regardless of mobility and AP's aspects such as MN's related direction with the AP and current AP's load.

### 4.7. Impact of MN's Number on Packet Loss Ratio

Figure 10(e) shows the packet loss ratio of AHP scheme, APCV, and D-Scan. The packet loss ratio has been normalized between 0 and 1. It can be noted that the AHP scheme obtained the lowest ratio, followed by APCV and D-Scan methods. As mentioned in the previous subsections, the AHP scheme achieved the lowest average handover delay in addition to low handover delay associated with real-time applications. For this reason, when MN performs the handover process with low delay, the connection is less likely to be broken during the handover procedure. Therefore, the probability of incurring a high packet loss ratio is low.

Furthermore, the impact of MNs increasing during simulation time on packet loss ratio is decreased by the proposed AHP scheme since load balancing is considered in the proposed adaptive fuzzy inference system. This is not surprising, since the packet loss ratio continued to decrease as the number of MNs increased, which implies that there are no handovers being processed by overloaded APs, which decreases packet loss ratio. From a different perspective, in order to place greater emphasis on the reasons behind the improvement in the ratio of packet loss utilizing proposed AHP scheme, Figure 10(f) presents the packet delay variation between calling and called party obtained by the AHP scheme.

Figure 10(f) illustrates the collected results of one example of two MNs communicating with each other by running a VoIP application type pulse-code modulation (PCM) with bit generation rate at 64 kbps. Throughout this example, the variance in time delay in delivering VoIP application packets is very similar between both the calling and the called party, where the calling party is the MN which initiates the call and the called party is the MN which answers the call. From the presented graphs which were collected during a handover process during simulation time, it can be observed that after 100 seconds the delay associated with the called party started to increase slightly as another handover process was started at that moment.

Afterwards, the delay increased when the packet ratio of VoIP increased during the calling session. After 100 seconds, however, the variation in the delay time between both the calling and the called parties was insignificant. Subsequently, after 490 seconds of simulation time, the second handover is started, and the variance in delay experienced between calling and called parties was in the range of 1 to 2 milliseconds.

### 4.8. Adaptive Fuzzy-Quality-Cost of Available APs in Simulation Scenario

In order to present the calculated Fuzzy-Q-Cost output of input metrics (RSS, *D*, and *L*), that is obtained with each run of adaptive fuzzy inference engine, one MN was selected in the simulation scenario. The calculated costs by the selected MN of all available APs using AHP scheme are presented in this subsection.  Figure 10(g) shows the scenario of the selected MN's movement trajectory (*Host1*) crossing 9 specific APs. The APs are sorted in the movement trajectory as sequence AP1, AP2, AP3, AP4, AP5, AP6, AP7, AP8, and AP9. Throughout these 9 APs, the obtained Fuzzy-Q-Cost by* Host1* illustrates that an average of 5 simulation runs are sufficient to serve as a numerical example of how to calculate cost in an AHP scheme.

Figure 10(h) illustrates the outputs of the proposed adaptive fuzzy inference engine which was employed in the AHP scheme to calculate the quality cost of AP1, AP2, and AP3. It is worth mentioning that the Fuzzy-Q-Cost of the APs was calculated every 10 seconds during the scanning process by* Host1* during its movement. As can be seen from Figure 10(h), the Fuzzy-Q-Cost ranges between 0 and 1 and is presented as a function of distance. At the first point of* Host1* movement (started its trajectory from AP1 as shown in Figure 10(g)), the obtained Fuzzy-Q-Cost is 1 (best quality cost) during the first 100 meters. Afterwards, the cost began to gradually decrease as the time distance was increasing, until sharply dropping to 0 beyond 800 meters.

The reason is that as the time distance increased between* Host1* and AP1, many quality aspects may have varied. For instance, when the MN is moving out of an AP's coverage area, the RSS will experience quality attenuation due to large scale fading. Moreover, direction can be changed to be more likely in low directed membership. Therefore, the obtained cost decreased as distance increased with AP1. On the other hand, the obtained costs from both AP2 and AP3 fluctuate by the increased distance during* Host1* movement. It is under such circumstances that the calculated cost, by employing proposed adaptive fuzzy inference system, can be either increased or decreased. For instance, as the load factors begin to vary between AP2 and AP3 with respect to two other input metrics (RSS and *D*), different costs result for AP2 and AP3 as plotted in Figure 10(h).

Figure 10(i) illustrates the obtained Fuzzy-Q-Cost for AP4, AP5, and AP6 during* Host1*'s movement trajectory as shown in Figure 10(g). As can be seen from the figure, at the first 100 meters of* Host1* movement, the Fuzzy-Q-Cost for AP4, AP5, and AP6 was low cost. This is not surprising since the allocated positions of the aforementioned APs in the movement trajectory are at a farther distance compared to AP1, AP2, and AP3. Therefore, the cost of AP4 and AP5 started to increase evenly by the time of* Host1* movement towards AP5 crossing AP4. Subsequently, the obtained cost of AP4 sharply decreased after 930 meters of* Host1* movement. In fact, two main AP4 ranking factors, which are RSS quality due to the movement out of AP4's coverage area and* Host1* moving in different direction with AP4 at this distance, are degraded. Therefore, the maximum achieved Fuzzy-Q-Cost for AP4 during simulation is a cost score of 0.635.

On the other hand, Figure 10(j) plots the obtained Fuzzy-Q-Cost of the last APs in* Host1* trajectory (AP7, AP8, and AP9) in the presented scenario in Figure 10(g). Based on the presented Fuzzy-Q-Cost graphs, the cost for these three APs was zero during the first 1200 meters of* Host1* movement, which increased the AP7 cost to 0.208 after 1220 meters. This sort of increase in the quality cost of AP7 fluctuated slightly between small cost values to a maximum value of 0.02. However, as the distance increased, the cost is increased as well, and, at 1400 meters, AP8 started to obtain small amounts of Fuzzy-Q-Cost as well.

The maximum Fuzzy-Q-Cost is obtained from AP7 which was 0.416 at 1910 meters of* Host1* movement. Beyond this distance, the calculated quality cost of AP7 started to gradually decrease. In this regard, it can be mentioned that based on defined* Host1*'s movement trajectory, the movement is adjusted close to the borders of AP7's coverage area. For this reason, as* Host1* moves farther distance away from AP7 after 1910 meters, the quality of two input metrics, RSS and Direction, is deducted. In the meantime, the Fuzzy-Q-Cost of AP8 and AP9 increased to a maximum achieved cost for AP8 of 0.98 at 1930 meters and the cost value of AP9 reached its maximum value (1) at 1980 meters.

Finally, the important point to note here is that throughout the obtained Fuzzy-Q-Cost as presented in the example of* Host1* all the available APs in MN's coverage area are ranked between 0 and 1 and are ready to choose the best candidate AP to camp on. Utilizing AP selection process as presented in Section 3.3, the best candidate AP can be chosen accordingly. Subsequently, all the associated delays in the handover process are decreased and the QoS of the ongoing applications are insured.

## 5. Conclusion

This paper proposed an adaptive handover prediction (AHP) scheme that predicts the best AP candidate considering the RSS of AP candidate, mobile node relative direction towards the access points in the vicinity, and access point load. As discussed in detail, the proposed AHP scheme relies on an adaptive fuzzy inference system to obtain predictions in the handover decision making process. This is achieved whereby coefficients are designed in a form and can be set up by the user. Afterwards, the membership functions of each particular input metric are calculated adaptively employing the developed piecewise linear equations. In the meantime, the weight vector for each input metric is proposed in an adjustable way to insure the accuracy of the obtained handover decision. Subsequently, the AHP scheme selects the final handover decision where AP obtained the highest quality cost (Fuzzy-Q-Cost), with respect to *h* which is the threshold value which helps to reduce unnecessary handovers. Simulation results show that proposed AHP scheme performs the best in contrast to the state of the art.

## Figures and Tables

**Table 1:** The Modified AP load element in beacon frame.

Octets				
1	1	2	1	2
AP_{load}	Length 7 octets	Station count	Channel utilization	Available admission capacity

**Table 2:** Knowledge structure based on fuzzy rules.

Rule	IF			THEN
	RSS	Direction	AP_load	AP-Q-Cost
1	Weak	Less-Directed	High	VLcost
2	Weak	Less-Directed	Medium	Lcost
3	Weak	Less-Directed	Low	Lcost
*⋮*	*⋮*	*⋮*	*⋮*	*⋮*
27	Strong	Medium-Directed	Low	VHcost

**Table 3:** Simulation parameters.

Parameters	Value
Simulation time	700 s
Simulation area	2500 × 1500 m
Mobility model	Rectangle and mass models
Number of MN	50
MN Speed	Maximum 60 km/h
Transmitted power WLAN	17 dbm
Transmission range of each AP	400 meter
Maximum packet generation rate	1350 packet/second
Maximum packet size	1000 byte
Channel bandwidth WLAN	11 Mbps
MAC protocol of WLAN	IEEE 802.11b PCF
